# Correlation of serum kisspeptin and leptin with non-obstructive azoospermia: A cross-sectional study in a subset of Karachi population

**DOI:** 10.12669/pjms.40.3.8125

**Published:** 2024

**Authors:** Sofia Amjad, Adnan Munir, Shamim Mushtaq, Rehana Rehman

**Affiliations:** 1 Sofia Amjad, Ph.D. Department of Physiology, Azra Naheed Medical College, Superior University, Lahore, Pakistan; 2 Adnan Munir, FCPS. Department of Andrology. Australian Concept Infertility, Medical Center Karachi, Karachi, Pakistan; 3 Shamim Mushtaq, Ph.D. Department of Biochemistry, Ziauddin University, Karachi, Pakistan; 4 Rehana Rehman, Ph.D. Department of Biological and Biomedical Sciences, Aga Khan University, Karachi, Pakistan

**Keywords:** Infertility, Kisspeptin, Leptin, Non-obstructive azoospermia, Spermatogenesis

## Abstract

**Objective:**

To determine the association of serum kisspeptin, leptin, and other hormonal profile with non-obstructive azoospermia (NOA) in infertile male subjects.

**Methods:**

This cross-sectional study was conducted at Australian Concept Infertility Medical Center, and Ziauddin University, Karachi from March 2018 to March 2020. The duration of the study was two years. Serum samples of 106 azoospermic participants were taken. Division of the subjects was done on a histological basis into obstructive azoospermia (OA) n=36, NOA n=70 which were further divided into spermatid maturation arrest (SMA), n=41, and sertoli cell-only syndrome (SCOS) n=29. Serum kisspeptin and leptin were measured by ELISA (Cloud-Clone Corp).

**Results:**

The follicle-stimulating hormone (FSH) (p<0.01), luteinizing hormone (LH) (p<0.01), thyroid-stimulating hormone (TSH) (p<0.01), and estradiol (p<0.01) was significantly high in the NOA group. However, kisspeptin was significantly decreased (p<0.01) in the NOA group. In the multivariate analysis after adjusting for other variables, the results showed that with the decrease in kisspeptin, the chances of being NOA were increased. Moreover, with the increase in Leptin, FSH, LH, and TSH the chances of being NOA were significantly enhanced.

**Conclusion:**

Serum kisspeptin, leptin, FSH, LH, TSH, and estradiol can be a potential marker for NOA in terms of better diagnosis, targeted therapeutic management, and the decision to proceed with surgical intervention.

## INTRODUCTION

Non-obstructive azoospermia (NOA) is the most challenging type of male infertility with limited treatment options and low success rates even with assisted reproductive techniques (ART). NOA is the absence of sperm in the ejaculate due to spermatogenic failure and affects 1% of the male population and 10-20% of infertile males.^1^ However, another type of azoospermia is obstructive azoospermia (OA) which is associated with normal spermatogenesis and obstruction in the seminal tract.^2^ Hormones have a pivotal role in the regulation of spermatogenesis by affecting sperm production, sperm quality, and steroidogenesis.^3^ Hormonal variations are one of the leading causes of NOA. Most of the time hormonal causes can be reversible if identified and empiric medical therapy is given.

**Fig.1:**
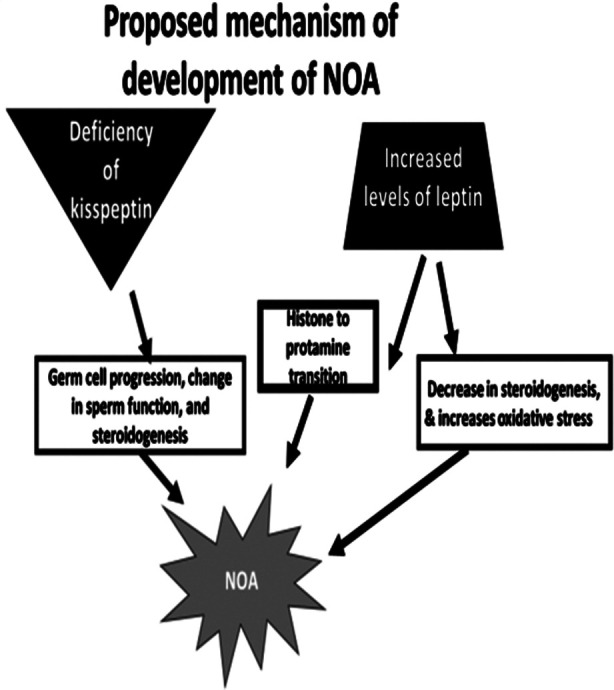
Proposed mechanism of development of NOA.

This could help couples avoid the stress of unnecessary therapies. Leptin is a hormone derived from adipocyte and plays a significant role in spermatogenesis through the hypothalamic-pituitary-gonadal axis (HPG axis) on gonadal hormone secretion.^4^ and maturation of spermatids from spermatocytes. However, when leptin levels exceed their physiological limit in obese men, it may have serious effects on male reproductive function such as inhibitory signal for testicular steroidogenesis, as well as on histone to protamine transition during spermatogenesis and oxidative stress^5^ Fig.1. High Leptin levels in the seminal fluid have been found to be correlated with decreased sperm motility and increased sperm DNA fragmentation.^6^

Kisspeptin (KISSI), a hypothalamic neuropeptide, supports male reproduction through regulation of the HPG axis through kisspeptin receptor (KISS1R) on hypothalamic gonadotrophins releasing hormone (GnRH) neurons.^7^ KISSI controls GnRH, gonadotrophins (LH, FSH), and sex hormones (testosterone and estradiol) secretion.^8^ KISSI has also a direct effect on the testes by the regulation of germ cell progression, sperm function, and steroidogenesis^9^ Fig.1. Any alteration in the central KISS1/KISS1R system may affect male fertility.^10^

Leptin’s effects on the reproductive system are mediated by kisspeptin neurons in the hypothalamus. This crosstalk of leptin- kisspeptin has also been seen in infertile males. Studies have been done on the association of KISSI and leptin with male infertility. However, there is a lack of data on the association of KISSI and leptin in NOA males and its mechanistic approach. Therefore, this study was designed to determine the association of serum kisspeptin, leptin, and other hormonal profile with NOA.

## METHODS

This cross-sectional study was conducted at Ziauddin University from March 2018 to March 2020. The duration of the study was two years. The sampling technique was purposive sampling. About 106 azoospermic patients who were coming for testicular sperm extraction in the Australian Concept Infertility Medical Center, enrolled after written informed consent. All the azoospermic males age range of 20 to 55 years and who have no sperm count in two consecutive semen analysis were included in the study. Patients with Klinefelter syndrome, Y chromosome deletion, and cystic fibrosis were excluded from the study.

### Ethical Approval:

It was obtained from the ethics review committee of Ziauddin University (No: 0020218, date: 26^th^ March 2018).

The medical history, physical examination, body mass index (BMI), body fat percentage (BF%), and andrological investigation were carried out. Azoospermia was confirmed by analyzing at least two semen samples that were concentrated by centrifugation. The BMI of the azoospermic patients was measured and categorized according to the “cut-offs for Asians, the normal weight (18-22.9 kg/ m2), overweight (23-24.9 kg/m2), and obesity (≥25 kg/m2)”.^11^,^12^ The BF% was measured by a bioelectrical impedance analyzer (BIA). The “cut-offs used for BF% were normal weight (12%-22%), overweight (22.1%-27%), and obese (>27.1%)”.^13^

The NOA patients were divided on a histological as the sample of testicular tissue was routinely sent for histological examination. Hence participants were categorized into obstructive azoospermia (OA) n=36, NOA n=70 which were further divided into spermatid maturation arrest (SMA), n=41, and Sertoli cell-only syndrome (SCOS) n=29.

Patient blood (5ml) was withdrawn by a trained laboratory phlebotomist using a sterile disposable syringe and was centrifuged, serum was separated and stored at -80ºC for hormonal assay. Serum kisspeptin was measured by (KISSI) ELISA Kit (Cloud-Clone Corp-CATALOG #: SEC559Hu) according to the manufacturer protocol. Serum leptin was measured by ELISA Kit (Cloud-Clone Corp- CATALOG #: SEA084Hu) according to the manufacturer protocol.

### Statistical analysis:

Data were analyzed using “Statistical Package for the Social Sciences (IBM-SPSS) version 23.0 and STATA version 12”. Descriptive for quantitative variables between three subgroups of azoospermic males: OA, SMA, and SCOS were assessed by using one-way ANOVA with a p-value of less than 0.05. “Strict Bonferroni correction” was done to get the significance of all the variables between the two subgroups by taking the p-value of less than 0.016. Univariate and multivariate analysis with their 95% confidence interval was done to estimate the relationship for NOA with all the study parameters by using “binary logistic regression”.

## RESULTS

Significant (p-value=0.016) decrease in age of the SCOS group of NOA. However, there were no significant results with other variables. Regarding BMI and BF were high in NOA groups, though results were not significant, (Table-I).

**Table-I:** Comparison of demographic variables among three subgroups OA, SMA, and SCOS of azoospermic males.

Demographic variables	OA (n=36)	Groups	

		NOA	

		SMA (n=41)	SCOS (n=29)
Age (years)	33.00±1.183	36.73±1.037	32.00±0.687^
Wife Age (years)	27.19±1.007	28.80±0.729	26.93±0.801
Height (meter)	1.76±0.020	1.80±0.025	1.78±0.022
Weight (kg)	81.75±3.22	79.15±2.52	80.24±2.83
BMI (kg/m^2^ )	26.50±1.064	24.52±0.772	25.41±0.946
BF (%)	21.19±1.25	22.800±1.037	22.55±1.261

Values are represented as Mean ± SE, Groups compared by ANOVA followed by Bonferroni OA, obstructive azoospermia; SMA, round spermatid maturation arrest; SCOS, Sertoli cell-only syndrome; BMI, body mass index. ^ p<0.016 was considered statistically significant for comparison of SMA & SCOS.

The mean of serum FSH (p-Value =0.016) was significantly high in the SCOS group as compared to the OA group and serum LH was found significantly high in SMA (p<0.001) versus OA group, SCOS (p<0.001) versus OA group, and SCOS (p<0.001) versus SMA group. High serum TSH was found significantly increased in SCOS (p<0.001) versus the OA group and SCOS (p=0.016) versus the SMA group. Significantly high levels of serum estradiol in SCOS (p=0.016) versus OA group and SCOS (p<0.001) versus SMA group. The mean of testicular volume was significantly decreased in SCOS (p=0.016) as compared to the OA group. Similarly, serum kisspeptin was significantly decreased in SMA (p=0.016) and SCOS (p=0.016)) as compared to the OA group. Serum leptin levels were high and serum testosterone was low in the SCOS group of NOA, however, the results were not statistically significant, (Table-II).

**Table-II:** Comparison of clinical Parameters among three subgroups OA, SMA, and SCOS of azoospermic males.

Clinical Parameters	OA N=36	NOA	

		SMA N=41	SCOS N=29
FSH (mlU /ml)	12.9 ±2.09	19.9 ± 2.98	28.10±3.11^#^
LH (mlU/ml)	4.80 ±0.16	7.02 ±0.24**	8.47 ±0.24^##^^^
TSH (mlU/L)	2.40±0.20	2.73±0.13	3.50±0.21^##^^
Estradiol (pg/ml)	26.70±1.49	19.03±0.98*	34.93±2.32#^^
Testosterone(ng/ml)	288.23±28.64	343.18±40.32	279.38±29.80
Testicular volume (ml)	21.95±4.83	14.21±0.340	7.181±0.223^#^
Kisspeptin (pg/ml)	216.78±28.31	133.30±11.62*	113.80±11.259^#^
Leptin (ng/ml)	2,214±0.410	3.04±0.460	3.618±0.597

Values are represented as Mean ± SE, Groups compared by ANOVA followed by Bonferroni. OA, obstructive azoospermia; SMA, round spermatid maturation arrest; SCOS, Sertoli cell-only syndrome; FSH, follicle-stimulating hormone; LH, luteinizing hormone; TSH, thyroid-stimulating hormone;

* p<0.016 was considered statistically significant for comparison of OA & SMA,

** p<0.001 was considered highly significant for comparison of OA & SMA, # p<0.016 was considered statistically significant for comparison of OA & SCOS, ## p<0.001 was considered highly significant for the comparison of OA & SCOS, ^ p<0.016 was considered statistically significant for comparison of SMA & SCOS, ^^ p<0.001 was considered highly significant for comparison of SMA & SCOS.

Univariate analysis and multivariate model (Table-III) after adjusting for other variables including age, wife age, and BMI, showed a decrease in kisspeptin, and an increase in Leptin, FSH, LH, and TSH the chances of being NOA increased.

**Table-III:** Association of NOA with Study Parameters using Binary Logistic Regression Analysis.

Parameters	Univariate Model (Un-Adjusted)	Multivariate Model (Adjusted ^a)^
	OR (95% C.I)	OR (95% C.I)
FSH	1.05* (1.02-1.08)	1.05* (1.02-1.08)
LH	4.43* (2.51-7.82)	4.88* (2.61-9.12)
TSH	1.74* 1.17-2.58)	1.73* (1.15-2.6)
Kisspeptin	0.99* (0.99 - 1)	0.9* (0.99-1)
Leptin	1.16 (0.99-1.35)	1.2* (1.02-1.41)
Sperm retrieval	21.2* (6.93-64.92)	37.6* (10.1-140.12)

^a^Model was adjusted for Age, wife Age, BMI C.I, confidence interval; FSH, follicle-stimulating hormone; LH, luteinizing hormone; TSH, thyroid-stimulating hormone;

* OR considered significant with p<0.05 by using Binary Logistic Regression Analysis.

## DISCUSSION

In our study, significant increases in FSH, LH, and TSH along with decreased testosterone levels in NOA as compared to the OA group have been observed. Comparable hormonal changes in NOA case has been documented in literature.^14^ The hypogonadism in NOA with a decreased production of testosterone causing an increase in FSH, LH by a negative feedback mechanism is responsible for this finding. Increase in levels of TSH has also been testified in infertile males^15^ which is based on reduced thyroid hormones effecting steroidogenesis, proliferation and differentiation of non-germ cells, and motility of sperm.^16^ High estradiol levels and obesity in the NOA group are similar to the outcomes of other researches in obese infertile males^17^ which is due to high aromatase activity causing conversion of testosterone to estradiol.^18^

Testicular volume is a significant clinical marker of hormonal as well as spermatogenic function. We have also found a decrease in testicular volume in the NOA group. This finding is also reported by Eelaminejad et al and others.^19^,^20^ Hence this reveals that with the decrease in testicular volume in NOA, the decrease in testosterone and spermatogenic function is evident. All these hormonal changes show primary hypogonadism and the extent of testicular pathology in NOA as compared to OA.^21^ Thus, hormonal derangement can be a useful marker of testicular damage causing a spermatogenic defect in NOA.

The current study has also shown significantly decreased serum levels of kisspeptin in NOA group. Other studies have also reported significantly decreased kisspeptin levels in infertile males and NOA.^10^,^22^ This study has revealed the direct effect of kisspeptin on testicular function by affecting steroidogenesis and spermatogenesis. Hence levels of kisspeptin can be used to access spermatogenesis in NOA. High leptin levels and obesity have been observed in NOA group in this study. However, the findings were not statistically significant. Hence this shows that obesity is causing high levels of leptin in the NOA group. Our study has also reported a decrease in testosterone and an increase in FSH and LH in this NOA subgroup. Similar findings have been reported by other studies in infertile males.^23^,^24^

These findings in NOA are due to the direct inhibitory effect of leptin on testicular steroidogenesis, on the transition of the histone to protamine during spermatogenesis, and oxidative stress causing an increase in DNA fragmentation, and a decrease in sperm count.^23^,^25^ It has also been found that the effects produced by leptin are reversible if the factors causing an increase in leptin are controlled.^26^ Leptin-Kisspeptin-fertility link has also been explored with female infertility in a maximum number of clinical pregnancies by Rehman et al.^27^ Thus, serum kisspeptin and leptin along with other hormonal profile should be estimated in infertile male subjects; a hope for NOA in terms of better diagnosis, targeted therapeutic management, and decision for the option of ART. Replacement therapy for hormonal deficiency of kisspeptin and lifestyle modifications to control leptin can be suggested for better fertility outcomes. Hence by treating the hormonal variations the costs, risks, and complications of assisted reproductive technologies (ART) which is the ultimate treatment for NOA men, can be reduced.

### Limitations:

There are certain limitations of our study. It is an unicentric study and hormonal levels of kisspeptin and leptin levels should have been checked in seminal plasma to see the direct effect. Further studies on a larger scale will be required to understand the pathophysiological role of kisspeptin and leptin in NOA males. In addition to this, follow-up studies should be done in the future to explore the therapeutic role of kisspeptin in the treatment of NOA.

## CONCLUSION

Variations in serum kisspeptin and leptin levels along with other hormonal profile is correlated with NOA. These variations consequently affect the spermatogenesis and lead to impairment of fertility. These hormones may be of great help in better diagnosis, targeted therapeutic management, and decision to proceed with surgical intervention.
